# Supplementary material for: A Global Screen for Assembly State Changes of the Mitotic Proteome by SEC-SWATH-MS
Source: Cell Syst. 2020 Feb 26;10(2):133–155.e6. doi: 10.1016/j.cels.2020.01.001 (PMC7042714; doi:10.1016/j.cels.2020.01.001)

Q9Y653 | AGRG1_HUMAN | ADGRG1 GPR56 TM7LN4 TM7XN1 UNQ540/PRO1083
Monomer MW [kDa]: 77.738 Monomer expected elution fraction: 42

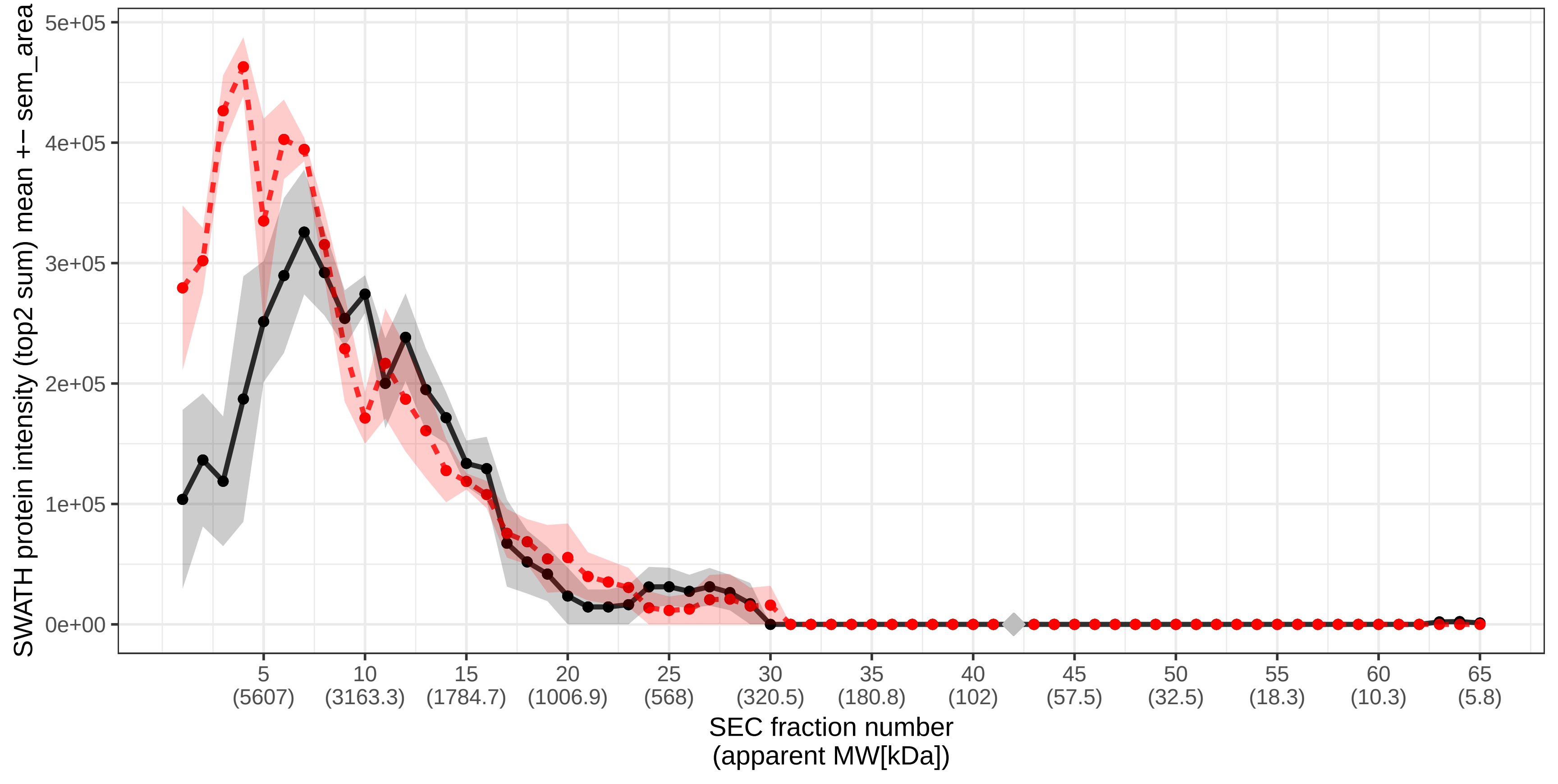

Supplement: Data S1. SEC-SWATH-MS Protein Chromatograms, Related to Figure 1 [file mmc6.zip › SECchrom_Q9Y653_AGRG1_HUMAN_ADGRG1_GPR56_TM7LN4_TM7XN1_UNQ5.pdf]
